# Dose-dependent modulation of hepatic cytochrome P450 enzymes by tenvermectin: implications for medication safety and combination therapy

**DOI:** 10.3389/fvets.2025.1647697

**Published:** 2025-08-25

**Authors:** Jianping Liang, Can Cui, Wenge Ren, Linglin Li, Linyi Lv, Xianhui Huang, Xiangmei Li

**Affiliations:** ^1^College of Food Science, Guangdong Provincial Key Laboratory of Food Quality and Safety, South China Agricultural University, Guangzhou, China; ^2^College of Veterinary Medicine, Guangdong Key Laboratory for Veterinary Drug Development and Safety Evaluation, South China Agricultural University, Guangzhou, China

**Keywords:** tenvermectin, cytochrome P450 enzymes, ivermectin, rats, inhibition and induction

## Abstract

**Introduction:**

Tenvermectin (TVM) is a novel avermectin-class drug that has attracted attention for its superior antiparasitic potency, low toxicity, and broad-spectrum activity. However, uncertainty about its interaction with cytochrome P450 enzymes (CYPs) has raised concerns about potential therapeutic failure, increased risk of toxicity, dangerous drug combinations, and prolonged discontinuation periods.

**Method:**

To address these critical safety concerns, we conducted a systematic comparative study using a highly selective and quantitatively accurate substrate conversion assay to assess and compare the effects of TVM and ivermectin (IVM) on the activities of key CYPs (CYP1A1/2, 2B1, 2C6, 2D2, and 3A1/2).

**Results:**

TVM induced CYP1A, 2C, 2D and 3A activities only at high therapeutic doses (2.5 mg/kg), and its induction was significantly weaker than that of IVM at all doses tested, with the most pronounced difference for CYP3A1/2. Although TVM had weak inhibitory effects on CYP2B1 and 2D2, at therapeutic concentrations these effects are presumably unlikely to cause clinically significant CYP-mediated drug interactions.

**Conclusion:**

As the first study to report the effects of TVM on CYP enzyme activity, these findings provide important experimental evidence and a theoretical framework for its clinical safety assessment, development of optimal dosing regimens, and rational polypharmacy strategies.

## 1 Introduction

Tenvermectin (TVM) is a novel avermectin-class drug produced by the genetically engineered bacterium *Streptomyces avermitilis* MHJ110 (1), which has a promising application in the treatment of gastrointestinal nematode infections and ectoparasites (2, 3). Compared with ivermectin (IVM), TVM has lower toxicity (3), rapid metabolism, and its area under the drug-time curve (AUC) and maximum plasma concentration (*C*_max_) are only 50% of those of IVM (4), and it has a better anthelmintic efficacy against *Ascaris suum* (pig roundworm) and *Trichuris suis* (pig whipworm) (3). As a drug candidate, systematic preclinical studies are essential to ensure the efficacy and safety of TVM in clinical applications (5). In particular, it is important to elucidate the mechanism of interaction between TVM and cytochrome P450 enzymes (CYPs) (6). Hepatic CYPs are involved in ~95% of drug metabolism (5, 7), and abnormalities in these enzymes during drug metabolism can lead to therapeutic failures, enhanced toxicity, risk of coadministration, and prolonged withdrawal times, ultimately jeopardizing efficacy, animal welfare, and food safety (7–10). However, no studies on the interaction of TVMs with CYPs have been reported to date. In addition, given the homology of CYP genes between rats and humans, dogs and pigs (9, 11), the study of the effects of TVMs on CYPs in rats may provide an important reference for predicting their rational use in other animal species.

Currently, a series of drugs similar to TVM have been reported in relevant studies: doramectin (0.3 mg/kg BW, subcutaneous injection (SC) or intramuscular injection) administered as a single dose has a low risk of inhibition of porcine hepatic CYPs (12). IVM is a moderate to weak inhibitor of human recombinant CYPs (CYP2C9, CYP2C19, CYP2D6, and CYP3A4) (13). However, the level of inhibition of CYPs by IVM at clinically recommended doses is unlikely to result in drug-drug interactions (DDI) (13). On the other hand, IVM significantly induced CYP1A activity in rats when administered at a dose 20–30 times the veterinary therapeutic dose (35 mg/kg BW, oral administration (PO), single dose), as well as CYP1A, CYP2B, and CYP3A1/2 activity in mouflon (0.5 mg/kg BW, PO, single dose) (14, 15). In humans, moxidectin (8 mg, PO, single dose) does not affect the activity of CYP3A4 (16). These studies indicate that despite their similar chemical structures, these drugs have different effects on the activity of CYPs due to dosage differences, different affinities with CYPs, and species differences (17). Therefore, in order to avoid the potential risk of DDI and promote the rational clinical application of TVMs, the results of similar drugs can only be used as a reference, and comprehensive and detailed experimental studies must be conducted for TVMs to obtain reliable data support. In addition, rat modeling studies may provide important insights to further explore the effects of TVM on CYPs in other species and help to reveal potential safety and efficacy issues.

**Scheme 1:**
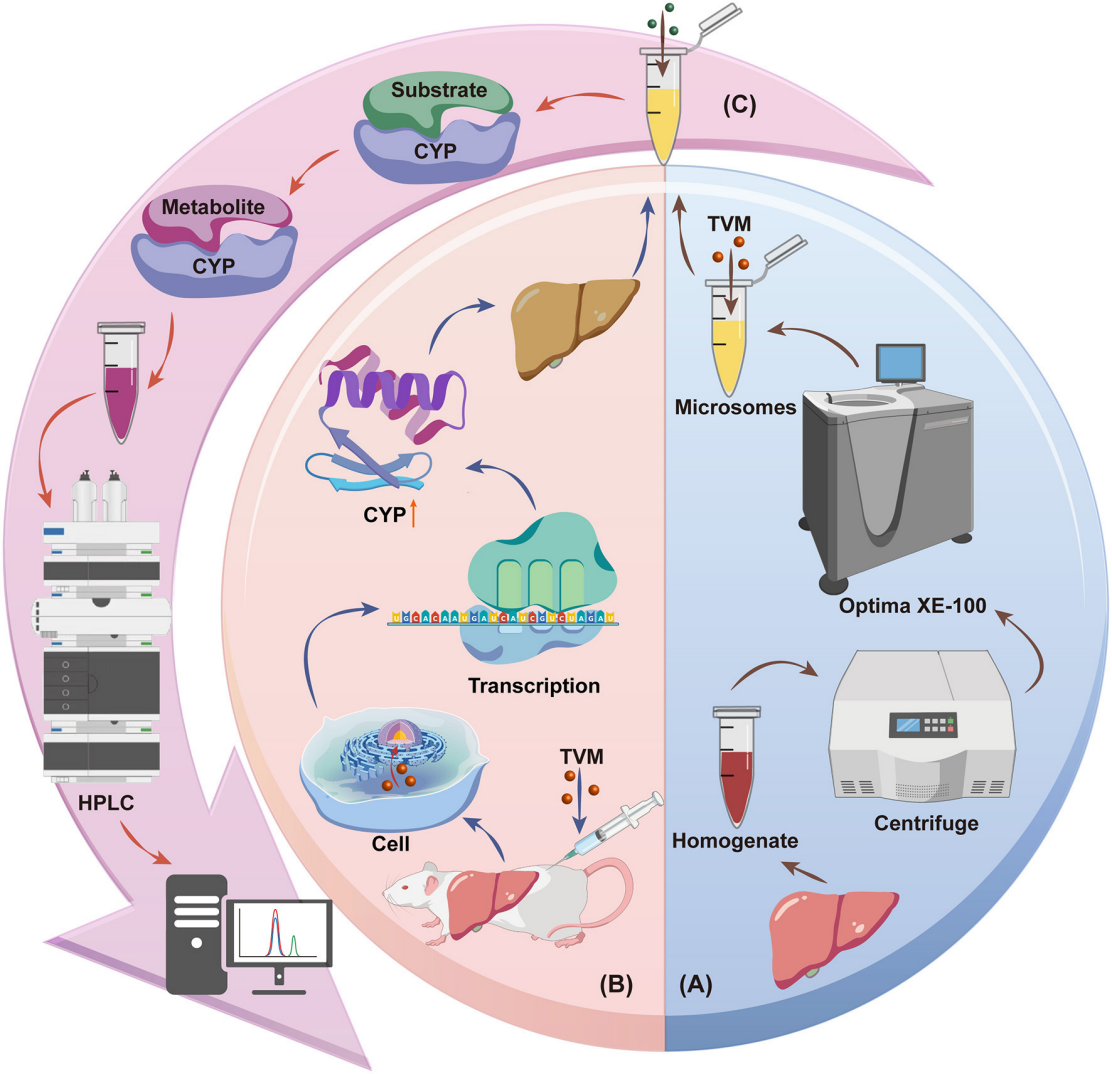
Schematic diagram of the effect of TVM on liver CYPs in rats: **(A)** Inhibition of CYPs by TVM, **(B)** Induction of CYPs by TVM, **(C)** Substrate conversion assay. Source: Created by the authors, using a generic diagram platform (GDP) by Jiang et al. (44).

In this study, we used a highly specific and quantitatively accurate substrate conversion assay to measure the activity of CYPs and determined the half-maximal inhibitory concentration (IC_50_) under *in vitro* to assess the inhibitory effect of TVM on the activity of CYPs (Scheme 1). In addition, we compared the changes in CYPs activity after SC of different doses of TVM to assess its induction of CYPs activity. IVM was used as an experimental control to further validate the effect of TVM on CYPs. These findings provide a scientific basis for the safe use of TVM in clinical practice and optimization of dosing strategies.

## 2 Materials and methods

### 2.1 Materials

TVM (98.3%) was provided by Tianwei Biopharmaceutical Co., Ltd. (Shenzhen, China). IVM (97.9%) was purchased from Qifa Drug Co., Ltd (Shandong, China). ER, resorufin (RF), diclofenac (DF), β-BNF, ketoconazole (KCZ), and quinine (QUI) were purchased from Yuanye Bio-Technology Co., Ltd. (Shanghai, China). DEX and α-BNF were purchased from Merck Sharp & Dohm (German). 4′-hydroxy diclofenac was ordered from Sigma Aldrich Corporation (USA). Dextromethorphan (DOM) and metyrapone (MTY) were purchased from Aladdin Holdings Group Co., Ltd. Benzyloxyresorufin (BR) was purchased from Saan Chemical Technology Co., Ltd. (Shanghai, China). Testosterone (TS), ordered from Ron Shanghai Lin'en Technology Development Co., Ltd. (Shanghai, China). Dextrorphan (DOR) was purchased from Quality Control Solutions (QCS) Standard Material Research and Development Center (Shenzhen, China). 6β-hydroxytestosterone (6β-OH-TS) was purchased from Weikeqi Biotechnology Co., Ltd. (Sichuan, China). PB-Na subscribed from Yanzhe Technology Co., Ltd. (Wuhan, China). All other chemicals and reagents were of analytical grade and commercially available.

### 2.2 Animal

Male SD rats, each weighing 250–320 g (11 weeks old), were purchased from the Laboratory Animal Management Center of Southern Medical University. The experiment was conducted at the Laboratory Animal Center of South China Agricultural University (2024-b134) following the guidelines for the care and use of laboratory animals issued by the National Science Council's Animal Center (18). Rats were euthanized by CO_2_ inhalation (flow rate: 30%−70% of chamber volume/min, no prefilled chambers). After respiratory arrest, CO_2_ was continued to be released for at least 1 min. To reduce hepatic glycogen content, no food was given for 24 h prior to euthanasia (18). The livers were then perfused with cold saline through the inferior vena cava to flush out the blood. The livers were harvested after the blood was thoroughly flushed out, and immediately frozen in liquid nitrogen. The liver tissues should be stored at −80°C.

### 2.3 Preparation of microsomes

The livers were washed two–three times in cold 0.05 M Tris-HCl solution (pH 7.4) and then homogenized with four volumes of homogenization buffer (0.25 M sucrose, 1 mM EDTA-2Na, and 0.05 M Tris-HCl solution) using a glass homogenizer kept in an ice bath. The resulting liver homogenate was centrifuged at 10,000 × *g* for 20 min at 4°C. The supernatant was further centrifuged in an ultracentrifuge (Optima XE-100) at 100,000 × *g* for 60 min at 4°C to obtain the liver microsomes as the pellet. The liver microsomes were then diluted to a concentration of liver weight to Tris-HCl buffer (1:1), aliquoted proportionally, and stored at −80°C for future use (19). The protein concentration of all samples was determined using a BCA Protein Assay Kit.

### 2.4 Substrate conversion assays

#### 2.4.1 Incubation conditions

The incubation system included: 0.05 M Tris-HCl buffer (pH 7.4), a certain concentration of liver microsomal protein, and an NADPH regeneration system (3.3 mM magnesium chloride, 0.05 mM sodium citrate, 3.3 mM glucose-6-phosphate, 1.3 mM NADP^+^, and 0.4 U/mL glucose-6-phosphate dehydrogenase). After pre-incubation for 3 min, the reaction was initiated by adding the respective substrate solutions (Table 1). Upon completion of the incubation, the corresponding stop solution was added to quench the reaction. For CYP2C6 and CYP3A1/2 incubation samples, IS [0.2 mM indomethacin (20), 0.4 mM carbamazepine (21)] were added. The samples were then vortexed for 5 min, followed by centrifugation at 4°C, 12,000 × *g* for 15 min. The supernatant was filtered through a 0.22 μm filter for instrumental analysis.

**Table 1:** Incubation conditions for each probe substrate.

**CYP isoform**	**Substrate**	**Metabolite**	**Protein concentration (mg/mL)**	**Incubation time (min)**	**Final solution**	**Internal standards**
CYP1A1/2	ER	RF	0.5	15	Methyl alcohol (v/v = 1:1)	None
CYP2B1	BR					
CYP2C6	DF	4′-OH-DF	0.5	10	3% carbinol formate (v/v = 1:1)	Indomethacin
CYP2D2	DOM	DOR	0.2	30	60% perchloric acid solution (v/v = 30:1)	None
CYP3A1/2	TS	6β-OH-TS	0.5	30	Methyl alcohol (v/v = 1:1)	carbamazepine

#### 2.4.2 HPLC and HPLC-MS/MS conditions

Summary of HPLC and HPLC-MS/MS methods for measuring various CYPs activities (Table 2). The HPLC methods for detecting CYP1A1/2 and CYP2B1 are based on the protocols described by Pegolo et al. (22). The method for assessing CYP2D2 activity is similar to those reported by Tian et al. (23) and Albassam et al. (24). Both methods were carried out on a Shimadzu LC-20A liquid chromatograph. The instrument is equipped with the following components: an LC-20AD quaternary pump, an RF-20A fluorescence detector, a DGU-20A5R degasser, a SIL-20ACXR autosampler, and a CTO-40C column oven. For CYP2C6, the HPLC system employed is the Shimadzu LC-20A liquid chromatograph. Samples are prepared by mixing with a mobile phase composed of water containing 0.1% formic acid and acetonitrile containing 0.1% formic acid, followed by injection into a C18 column for gradient elution at a flow rate of 0.3 mL/min. Analysis is performed using a Shimadzu LCMS-8045 triple quadrupole mass spectrometer with an electrospray ionization (ESI^+^) source in multiple reaction monitoring mode. The transitions monitored include *m*/*z* 266.0 → 231.0 (CE = −13 eV) for 4'-OH-DF and *m*/*z* 195.0 (CE = −19 eV) for indomethacin as IS. For CYP3A4, the HPLC-MS/MS system comprises an Agilent 1200 Series liquid chromatograph coupled with an API 4,000 triple quadrupole mass spectrometer. The mobile phase is delivered at a flow rate of 0.5 mL/min for gradient elution. Mass spectrometric analysis is conducted in multiple reaction monitoring mode under positive ion conditions, with the ion spray voltage set to 5,500 V and the ion source temperature maintained at 550°C. The collision gas, curtain gas, nebulizer gas, and auxiliary gas are set to 12, 35, 55, and 55 psi, respectively. The qualitative and quantitative ions for 6β-OH-TS are *m*/*z* 287.4 → 269.2 (CE = 20/19 eV), and the Q3 transition for the carbamazepine is *m*/*z* 195.1 (CE = 25 eV).

**Table 2:** HPLC and HPLC-MS/MS conditions for the various analyses of the probe substrate reactions.

**Assay**	**Solvents^a^**	**Column^b^**	**Flow rate (mL/min)**	**Gradient^c^**	**Total run time (min)**	**Detection (nm)**	**Mass transition**	**Instrument^d^**
RF	A, B, C	G	1.0	A/B/C, 52/45/3	8	λ_EX_ = 560 nm, λ_Em_ = 586 nm	None	K
4′-OH-DF	D, E	H	0.3	1–2 min, 30%−70% B; 5.5–6 min, 70%−30% B	7	None	312.0 → 231.0/266.0, (IS) 358.0 → 139.0	L
DOR	F, C	L	1.0	8–9 min, 15%−25% B; 18–19 min, 25%−15%	22	λ_EX_ = 280 nm, λ_Em_ = 330 nm	None	K
6β-OH-TS	D, C	J	0.5	1–1.5 min, 25%−19% B 5.2–5.3 min, 19–32% B 13–14 min, 32%−25% B	16	None	305.1 → 269.2/287.4, (IS) 238.0 → 195.1	M

^a^A, 20 mM phosphate buffer (pH 6.8); B, methanol; C, acetonitrile; D, 0.1% formic acid in water; E, 0.1% formic acid in acetonitrile; F, 1.5% glacial acetic acid.

^b^G, Phenomenex Luna C8 (4.6 × 150 mm, 5 μm); H, Phenomenex Titank C18 (50 × 2.1 mm, 1.8 μm); L, Kromasil 100-5-C8, (4.6 × 150 mm, 5 μm,); J, Phenomenex luna^®^ (150 × 2 mm, 5 μm).

^c^All gradient segments were linear, generally, with a 0.5- to 1-min gradient to starting conditions and 2–9 min of re-equilibration.

^d^K, Shimadzu LC-20A liquid chromatograph; L, Shimadzu LCMS-8045 triple quadrupole mass spectrometer; M, Agilent 1200 Series liquid chromatograph and APl 4000 triple quadrupole mass spectrometer.

#### 2.4.3 Methodological validation

According to the guidelines for bioanalytical method validation of the US Food and Drug Administration, the methodological validation was performed for the above four analytical methods (selectivity, calibration curve, carryover, sensitivity, accuracy and precision, recovery, and stability). Accuracy and precision were obtained using a linear equation from six-point calibration. Stability encompasses stock solution stability, autosampler stability and short-term stability (25). When testing samples, each batch is accompanied by calibration curves and quality control samples at high, medium, and low levels to ensure the stability of the instrument.

### 2.5 Inhibition of CYPs by TVM and IVM

To determine the inhibitory potential (IC_50_) of TVM and IVM on the activity of CYPs (CYP1A1/2, 2B1, 2C6, 2D2, and 3A1/2), substrates at concentrations approximately equal to their respective *K*_m_ values were mixed with different concentrations of TVM or IVM (0.5–150 μM), with the total organic solvent content not exceeding 1%, and then incubated according to the microsome protein concentration and incubation time in Table 1. The blank control group was replaced with an equal volume of methanol and TVM or IVM was added, and corresponding positive groups were designed: α-BNF (CYP1A1/2), MTY (CYP2B1) (26), QUI (CYP2D2), and KCZ (CYP3A1/2) (27).

### 2.6 Induction of CYPs by TVM and IVM

To investigate the induction of CYPs by TVM, low, medium (equivalent to the standard veterinary therapeutic dose), and high doses of TVM or IVM were administered and subsequent changes in the activity of liver microsomal CYPs were monitored (28). Rats were randomly divided into 12 groups and five rats in each group. These groups were Blank, TVM-0.5, TVM-1.25, TVM-2.5, IVM-0.5, IVM-1.25, IVM-2.5, Oil, Salt, β-BNF, PB-Na, and DEX. The blank control group was a mixture of propylene glycol, PEG 4000, and water (the blank solvent for TVM and IVM); TVM-0.5, TVM-1.25, and TVM-2.5 groups were administered with a single SC at a dose of 0.5, 1.25, and 2.5 mg/kg BW; the same was true for IVM-0.5, IVM-1.25, and IVM-2.5 groups. β-BNF, PB-Na, and DEX are all chemical inducer groups: β-BNF (CYP1A1/2) dissolved in corn oil solution, 80 mg/kg BW intraperitoneal injection (IP), once a day, for 3 days (29); PB-Na (CYP2B1 and CYP2C) dissolved in normal saline solution, 80 mg/kg BW, IP, once a day, for 4 days (30); DEX (CYP3A) dissolved in corn oil solution, 100 mg/kg BW, IP, once a day, for 4 days (31).

### 2.7 Data processing

Michaelis-Menten kinetic data, including *K*_m_ and *V*_max_, were fitted by nonlinear regression analysis using GraphPad Prism 9, and Eadie-Hofstee plots were obtained to determine the kinetic type. The enzyme inhibition function of the dose-response was used to calculate the IC_50_. All data were obtained from triplicate reactions and are expressed as mean ± standard deviation. Two-tailed Student's *t*-test was used to determine the significance of differences in CYPs activity after drug treatment. *p* < 0.05 was considered significant, *p* < 0.01 was very significant, and *p* < 0.001 was extremely significant.

## 3 Results

### 3.1 Substrate conversion assays

In order to quantitatively assess the effects of TVM on the activities of key CYPs, HPLC and HPLC-MS/MS analytical methods based on the principle of substrate conversion assay were developed for the quantitative determination of the metabolites (resorufin, 4'-hydroxydiclofenac, dextrorphan and 6β-hydroxytestosterone). Specifically, the reaction rates of ethoxyresorufin O-deethylation (EROD) and benzyloxyresorufin O-dealkylation (BROD), diclofenac 4'-hydroxylation (DFH), dextromethorphan N-demethylation (DMOD), and testosterone 6β-hydroxylation (T-6β-OH), were used as indicators for assessing the activity of CYP 1A1/2, 2B1, 2C6, 2D2, and 3A1/2, respectively.

The results of methodological validation (Tables 3, 4 and Supplementary Figure S1–S4) showed that the constructed method could effectively separate the target analytes (metabolites), internal standards (IS) and endogenous components in the matrix with high sensitivity, which satisfied the requirements of the determination; the linearity was good within the set concentration range (*R*^2^ > 0.99); and the residual effect did not interfere with the accuracy and precision. The deviation of accuracy (DEV) ranged from −14.83% to 13.05%, the coefficient of variation (CV) ranged from 1.10% to 8.29%, and the extraction recoveries ranged from 88.75% to 107.30%, which enabled the precise quantification. Under the analytical conditions in Table 4, the stability of the methods was good and suitable for the analysis of real samples, which fully verified their reliability and accuracy.

**Table 3:** HPLC and HPLC-MS/MS method's selectivity, calibration curve, sensitivity, carryover, accuracy, precision, and recovery.

**Assay**	**Selectivity (%)**	**Calibration curve**		**Sensitivity**		**Carryover (%)**	**Accuracy and precision**		**Recovery (%)**
		**The equations of the calibration curve**	*R* ^2^	**LOQ (nM)**	**LOD (nM)**		**The intra- and intre- accuracy bias (DEV %)**	**The intra- and intre- precision (CV %)**	
RF	0	*y* = 2,835.53*x* – 42.6690	0.9994	5	1	< 2.44	−5.86 to 11.08	1.10–6.05	99.36–107.30
		*y* = 3,569.48*x* + 1,341.96	0.9934						
		*y* = 2,437.43*x* + 729.183	0.9988						
4′-OH-DF	< 1.09; < 0.21 (IS)	*y* = 0.00727758*x* + 0.0134207	0.9938	30	25	< 4.27; < 0.15 (IS)	−14.83 to 13.05	1.47–6.74	97.13–97.32; 98.52 (IS)
		*y* = 0.00679554*x* + 0.0242021	0.9974						
		*y* = 0.00728104*x* + 0.0149169	0.9982						
DOR	0	*y* = 170380*x* – 1258.06	0.9959	150	60	0	−8.53 to 6.94	1.33–4.21	93.40–97.92
		*y* = 178311*x* – 2833.61	0.9961						
		*y* = 182237*x* – 3329.98	0.9971						
6β-OH-TS	< 2.88; < 0.02 (IS)	*y* = 0.795*x* + 0.00209	0.9966	300	150	< 2.71; < 0.53 (IS)	−4.56 to 10.39	1.34–8.29	88.75–99.96; 103.86 (IS)
		*y* = 0.72*x* + 0.00677	0.9960						
		*y* = 0.732*x* + 0.00154	0.9920						

**Table 4:** The stability of HPLC and HPLC-MS/MS method.

**Assay**	**Stock solution stability (%)**	**Autosampler stability (DEV %)**	**Short-term stability (DEV %)**
RF	113.15 (4°C, 7 days)	−7.98 to 2.37 (24 h)	−4.26 to 2.86 (2 h)
4′-OH-DF	90.83; 103.25 (IS) (−80°C, 30 days)	−11.41 to 7.63 (18 h)	1.50 to 12.75 (2 h)
DOM	108.42 (−80°C, 30 days)	−7.60 to 3.36 (24 h)	−3.60 to 11.82 (2 h)
6β-OH-TS	111.22; 98.39 (IS) (−20°C, 30 days)	9.61 to 8.06 (24 h)	8.44 to 2.67 (2 h)

**Figure 1:**
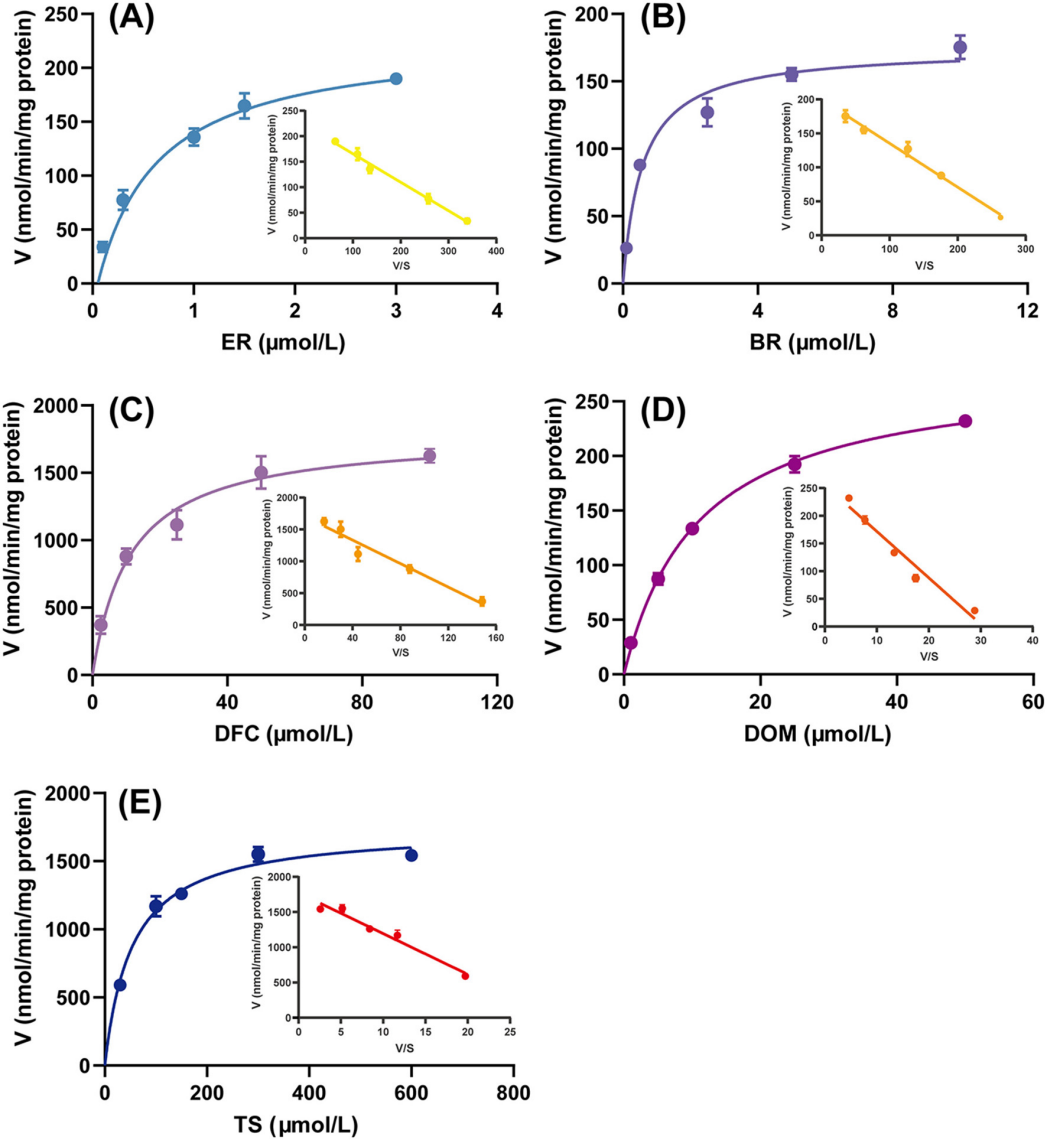
Michaelis-Menten kinetic data and Eadie-Hofstee plots for the metabolism of **(A)** ethoxyresorufin (ER) (CYP1A1/2), **(B)** benzyloxyresorufin (BR) (CYP2B1), **(C)** diclofenac (DF) (CYP2C6), **(D)** dextromethorphan (DOM) (CYP2D2), and **(E)** testosterone (TS) (CYP3A1/2) to resorufin (RF), 4′-hydroxydiclofenac (4′-OH-DF), dextromethorphan (DOR), and 6β-hydroxytestosterone (6β-OH-TS), respectively, in rat liver microsomes. Data are expressed as mean ± standard deviation, *n* = 3.

The Michaelis–Menten kinetic model is the classic theoretical model describing the relationship between the rate of an enzymatic reaction and the substrate concentration. Eadie-Hofstee plots are commonly used to estimate *K*_m_ and *V*_max_ from experimental data, and when the plots show a linear trend, it implies that the reaction is catalyzed by a single enzyme rather than by the synergistic action of multiple enzymes (32). As shown in Figure 1, the enzyme kinetic curves of the five CYP isozyme reactions saturated with increasing substrate concentration, and the corresponding Eadiehofstee plots were all linear, indicating that these reactions are simple single-substrate-single-product reactions that can accurately reflect the activities of the corresponding CYPs.

### 3.2 Inhibition of CYPs by TVM and IVM

**Figure 2:**
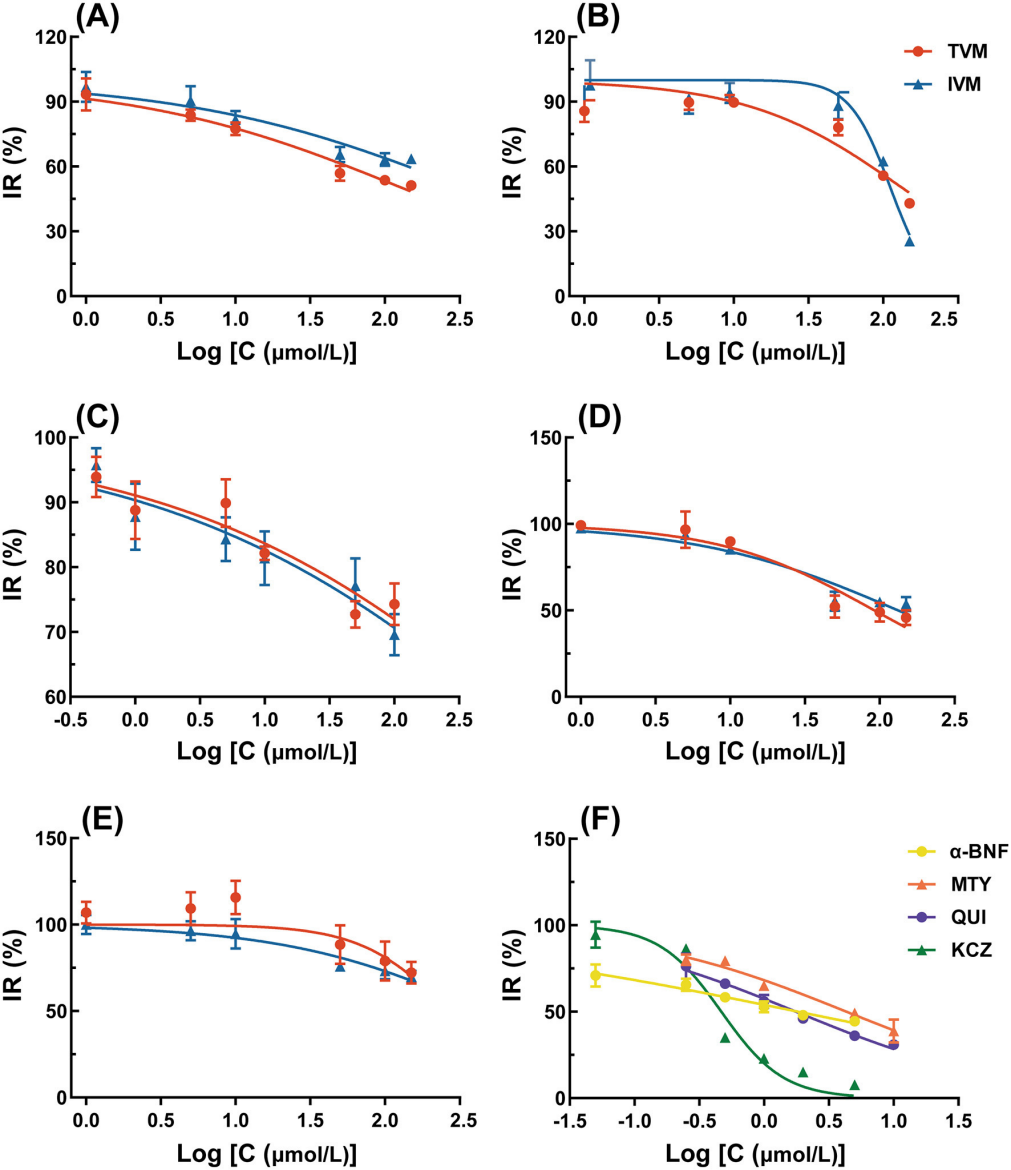
Enzyme inhibition rates of TVM and IVM for five rat CYPs: **(A)** CYP1A1/2, **(B)** CYP2B1, **(C)** CYP2C6, **(D)** CYP2D2, **(E)** CYP3A1/2; and **(F)** Inhibition rates of four positive inhibitors for four rat CYPs: α-naphthoflavone (α-BNF; CYP1A1/2), metyrapone (MTY; CYP2B1), quinine (QUI; CYP2D2), ketoconazole (KCZ; CYP3A1/2). The inhibition rate is expressed as relative activity compared to the control group. Data are expressed as mean ± standard deviation, *n* = 3.

The inhibition function curves of TVM and IVM on the activity of CYPs at different doses are shown in Figure 2, and the IC_50_ values obtained are listed in Table 5. The results showed that the IC_50_ values were above 100 μM (Figures 2A, C, E), which indicated that TVM and IVM had no inhibit CYP 1A1/2, CYP 2C6, and CYP 3A1/2. According to the inhibition law of drugs on the activity of CYPs, the lower IC_50_ value means the stronger inhibition effect. In this theoretical framework, both drugs showed a slight inhibitory effect (50 μM < IC_50_ < 100 μM) against CYP2B1 (Figure 2B), with IC_50_ values of 72.30 and 58.64 μM, respectively. For CYP2D2, although the inhibitory function curves of TVM and IVM were similarly obtained (Figure 2D), the TVM showed a weak inhibitory effect IC_50_ = 91.93 μM. To verify the reliability of the experiment, the positive control group showed strong inhibition in all tests (IC_50_ < 10 μM, Figure 2F). This result provides strong evidence that the experimental system is capable of accurately and reliably assessing inhibition of the target CYPs and ensures the validity of the TVM and IVM inhibition data.

**Table 5:** CYPs inhibition by TVM and IVM *in vitro*.

**CYP isoform**	**IC**_**50**_		
	**TVM**	**IVM**	**Positive control**
CYP1A1/2	>100	>100	1.82 (1.35–2.63) (α-BNF)
CYP2B1	72.30 (53.29–108.2)	58.64 (46.38–71.28)	4.31 (3.46–5.55) (MTY)
CYP2C6	>100	>100	None
CYP2D2	91.93 (70.78–125.10)	>100	1.76 (1.51–2.06) (QUI)
CYP3A1/2	>100	>100	0.47 (0.39–0.56) (KCZ)

Data are expressed as IC_50_ (μM) (range), (*n* = 3).

### 3.3 Induction of CYPs by TVM and IVM

**Figure 3:**
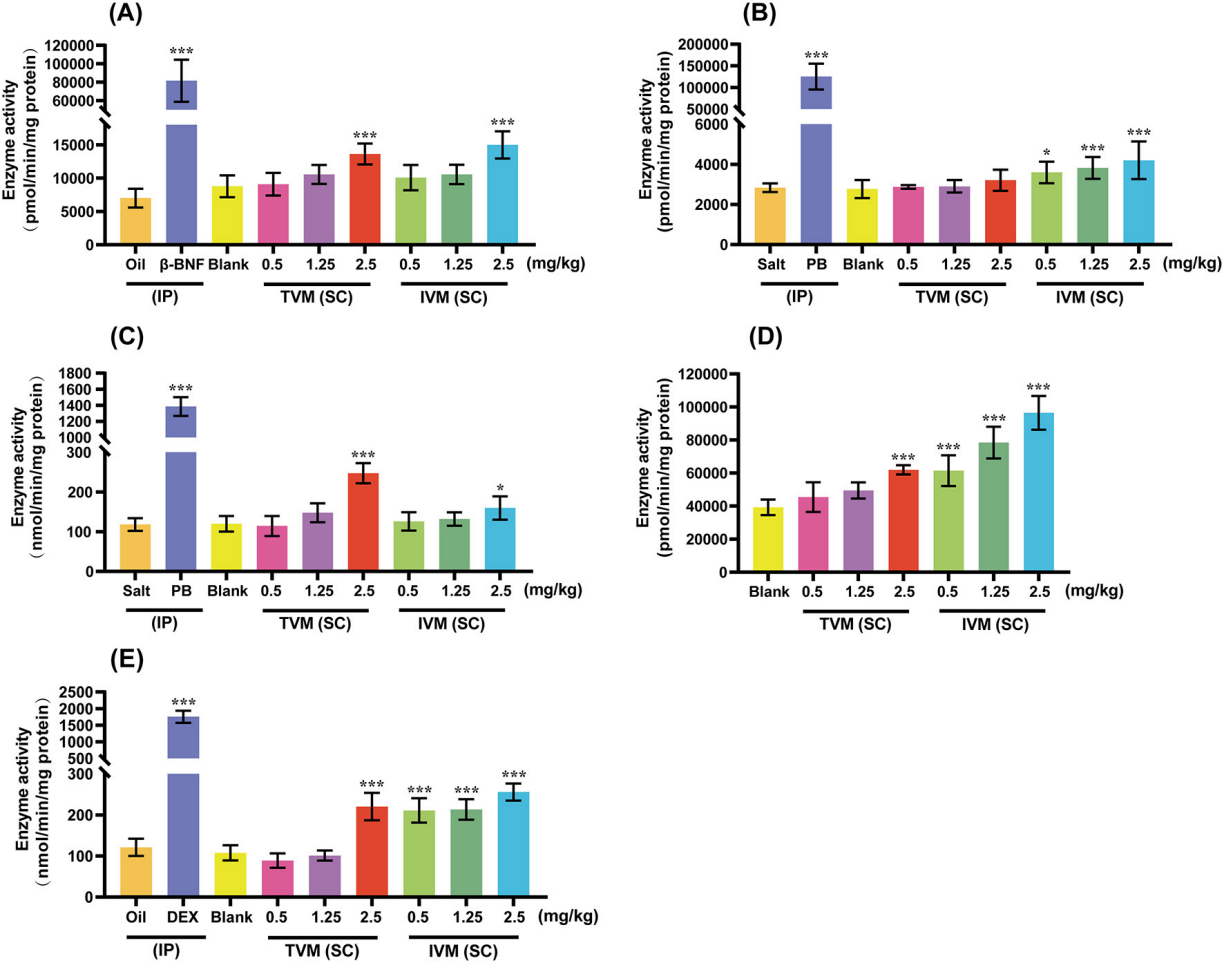
The induction effects of TVM and IVM on CYP1A1/2 **(A)**, CYP2B1 **(B)**, CYP2C6 **(C)**, CYP2D2 **(D)**, and CYP3A1/2 **(E)** in rat liver. TVM/IVM was administered at single doses of 0.5, 1.25, and 2.5 mg/kg BW (SC), β-naphthoflavone (β-BNF) at 80 mg/kg BW/day for 3 days (IP), Phenobarbital Sodium (PB-Na) at 80 mg/kg BW/day for 4 days (IP), and dexamethasone (DEX) at 100 mg/kg BW/day for 4 days (IP). Inhibition rate is relative to enzyme activity, calculated as 100% of control activity. Each value represents the mean ± standard deviation of five rats. *p < 0.05; ***p < 0.001, TVM/IVM vs. Blank; Positive control vs. Oil/Salt. Due to the lack of a clinically recognized or mechanistically clear CYP2D inducer, no positive control group was set up for CYP2D2 induction.

In the present study, we systematically evaluated the potential of drug candidates to induce CYP activity by comparing enzymatic activity levels across experimental, blank, and positive control groups (Figure 3). The positive control group (β-BNF, PB-Na, DEX) increased the activities of CYP1A1/2, CYP2B1, CYP2C6, and CYP3A1/2 by 11.64-, 44.18-, 11.71-, and 14.50-fold, respectively, as compared with the solvent control (corn oil/saline). The obvious activity-inducing effect verified the validity and reliability of the animal experiments.

The induction of CYPs activity by IVM showed a clear dose-dependent and isoform-selective effect. As shown in Figure 3, different doses of IVM significantly increased the activities of CYP2B1 (1.30-, 1.38-, and 1.52-fold), CYP2D2 (1.56-, 2.00-, and 2.46-fold), and CYP3A1/2 (1.96-, 1.98-, and 2.37-fold). High-dose IVM (2.5 mg/kg BW) also induced other CYPs to varying degrees: CYP1A1/2 (1.70-fold) and CYP2C6 (1.33-fold). In summary, IVM had the most pronounced induction of CYP3A1/2, followed by CYP2D2 and CYP2B1, and relatively weak induction of CYP1A1/2 and CYP2C6. The weaker induction of CYPs by the drug candidate TVM reduced the risk of DDI compared to IVM. CYPs (CYP1A, 2C, 2D, and 3A) activity was induced by TVM only at high doses, with 1.55-, 2.06-, 1.57-, and 2.04-fold increase in enzyme activity, respectively, compared to the blank control (Figure 3).

## 4 Discussion

In drug metabolism studies, EROD, BROD, DFH, DMOD, and T-6β-OH are the classic substrate probes for the assessment of CYPs activity. It has been shown that in the rat liver metabolic system, CYP1A1/2 is the predominant isoenzyme affecting EROD activity (Figure 1A), although other CYPs may be involved in the process, but with relatively low contributions (33). For the BROD reaction, although it is commonly used to assess CYP2B1 activity, CYP1A1/2 also catalyzing the reaction can confuse the assessment of CYP2B1 activity (26). To solve this problem, the optimized incubation system significantly improved the accuracy of CYP2B1 activity determination by adding 5 μM α-BNF to specifically inhibit the CYP1A1/2-mediated metabolic pathway (Figure 1B). In addition, DFH, DMOD and T-6β-OH have been shown to be predominantly catalyzed by CYP2C6, CYP2D2, and CYP3A1/2, respectively (34). The specificity of these substrate probe reactions for the characterization of the corresponding CYPs activities was further validated by the linear characterization of the Eadie-Hofstee plots (Figure 1) in this study.

From a clinical risk perspective, inhibitory effects of drugs on CYPs are usually more potentially harmful than inducible effects (35), as they can reduce drug metabolism and lead to enhanced potency or adverse effects (36). Previous studies have shown that IVM moderately inhibits human recombinant CYP 2C9, 2C19, 2D6, and 3A4 (13), but in the present study, we found that IVM only weakly inhibited rat CYP2B1 (IC_50_ = 58.64 μM). Notably, the *C*_max_ of IVM under different routes of administration (SC, intramuscular injection, intramuscular injection, and PO) in a variety of animals (cattle, sheep, pigs, canines, and horses) were lower than 150 ng/mL (~0.2 μM) (37). Similarly, TVM although weakly inhibiting CYP2B1 and CYP2D2 (IC_50_ of 72.30 and 91.93 μM, respectively), had a *C*_max_ of only 245.89 ng/mL (~0.3 μM) in rats (unpublished data from our laboratory), again suggesting a low risk of inhibition *in vivo*.

In the field of research on the induction of CYPs activity by avermectins, no relevant studies have been reported on the effect of TVM and IVM on the enzyme activity of CYPs after SC. Previous studies have shown that when rats were administered IVM (35 mg/kg BW, PO) at doses 20–30 times the veterinary therapeutic dose, EROD activity significantly increased, while a dose of 0.3 mg/kg BW had no effect on this activity (14). Following a single dose of IVM (0.5 mg/kg BW, PO) in mouflons, the activity of CYP1A, 2B, and 3A1/2 significantly increased, while the activity of CYP2E1 remained unchanged (15). In this study, the induction of rat CYPs by IVM also exhibited a similar dose-dependent pattern (Figure 3). At a dose of 2.5 mg/kg BW, IVM significantly induced the activity of CYP1A, 2B1, 2C6, 2D2, and 3A1/2, while the two lower doses had no significant effect on the activity of CYP1A and 2C6. However, the induction effect of TVM was much lower than that of IVM, with weak induction of CYP1A, 2C, 2D, and 3A only at high doses, suggesting that the problem of attenuation of efficacy of conventional avermectin analogs due to self-induction of the CYPs can be circumvented. The reduced induction effect may be due to the weaker lipophilic structure of TVM than IVM (4), its shorter accumulation time in adipose tissue and higher plasma clearance (13, 14), which in turn reduces the possibility of TVM's induction of CYPs. The reasons for this need to be further studied.

The strong induction of CYP3A by IVM further exacerbates the clinical risk of drug use. As a key CYP enzyme involved in the metabolism of ~60% of human drugs (38), up-regulation of CYP3A activity may accelerate the metabolism of co-administered drugs, triggering widespread DDI, and, according to Barber, S. et al., the combination of IVM (SC) and moxidectin (whose main metabolizing enzyme is CYP3A) produced higher AUC in sheep than moxidectin alone, a phenomenon likely related to the relatively high 6β-TOH activity in the sheep microsomes (39). From this point of view, TVM should be more suitable for combination therapy with moxidectin than IVM. On the other hand, IVM show better suitability when combined with anthelmintics that have metabolites as their main pharmacodynamic effect, albendazole being a typical example [CYP3A4 is a key factor in the formation of albendazole sulfoxide (40)]. In a report by Alvarez et al. (41), the combination of albendazole + IVM (SC) was compared with albendazole administered alone to lambs, and a 40% increase in the AUC of albendazole sulfoxide was found in the former compared to the latter. Taken together, it is likely that the induction of CYP3A by IVM (SC) is responsible for the increase in plasma levels of albendazole sulfoxide in sheep. In addition, anthelmintic treatment with a combination of IVM and albendazole may also be a more appropriate option for some animals with high intrinsic CYP 3A activity, such as miniature pigs (42).

On the other hand, the induction of CYP1A1/2, which is mainly responsible for catalyzing the biotransformation of organic environmental pollutants (which include pro-carcinogens such as PAHs and PCBs), is also associated with the activation of the protein kinase cascade as well as with the increase of mitosis and cellular proliferation, a complex with toxic effects (14). In the present study, a weak induction of CYP1A1/2 activity was observed using both IVM (SC) and TVM (SC) at two-fold veterinary doses. Of interest, the EROD activity of bovine microsomes was five-fold higher than that of microsomes from other farm species (e.g., pigs, goats, and sheep) (42). Based on this difference, when treating cattle with IVM and TVM, high-dose regimens should be carefully selected and utilized in order to effectively reduce the potential risk of mutagenicity and carcinogenesis. In addition, special care should be taken to avoid combining IVM and TVM with drugs metabolized by CYP1A, such as estradiol, during clinical treatment (42).

In addition to the potential for DDI in the treatment of domestic animals, IVM administered via SC may have some impact in companion animal applications. In canines, the clinical substrates of CYP 2B and CYP 2D have been relatively well studied and described. Canine CYP 2B plays an important role in drug metabolism by facilitating the bioclearance process of cyclophosphamide (a commonly used chemotherapeutic drug) and propofol (an anesthetic drug) (9, 43). Meanwhile, CYP 2B is also involved in the metabolism of various endogenous substrates (e.g., androstenedione and progesterone) as well as toxic environmental chemicals (e.g., polychlorinated biphenols) (43). CYP 2D, on the other hand, possesses the ability to catalyze the biotransformation of antiemetic drugs (e.g., maropitant, metoclopramide) in addition to its ability to efficiently scavenge a number of antidepressants (e.g., clomipramine, fluoxetine) and opioid derivatives (e.g., tramadol) (43). This implies that the effects of IVM on CYP2B and CYP2D, as well as possible interactions with other drugs, need to be taken into account when treating canine companion animals, so as to formulate a more scientific and rational therapeutic regimen and to ensure the safety and efficacy of the medication.

## 5 Conclusion

This study is the first to systematically assess the effect of TVM on the activity of CYPs. The study showed that the inhibitory effect of TVM on CYPs is very unlikely to occur *in vivo*, and that the induction of CYPs by TVM was dose-dependent and significantly weaker than that of IVM, with a more pronounced induction of CYPs only at doses up to twice the usual veterinary dose. The induction of CYPs is significantly weaker with TVM than with IVM—only at doses up to twice the usual veterinary dosage. Therefore, TVM is feasible for combination therapy with most drugs. These findings validate the superior clinical safety and therapeutic potential of TVM, provide key evidence in support of its metabolic properties, and lay a solid foundation for subsequent translational clinical studies.

## Data Availability

The original contributions presented in the study are included in the article/Supplementary material, further inquiries can be directed to the corresponding authors.

## References

[1] Zhu LY Zhang SY Lu F Zhang K Han QQ Ying QW. Cross-resistance, fitness costs, and biochemical mechanism of laboratory-selected resistance to tenvermectin A in *Plutella xylostella*. Pest Manag Sci. (2021) 77:2826–35. 10.1002/ps.6317 33538385

[2] Wan X Zhang SY Zhang H Zhai J Huang J Chen AL. Two new tenvermectins from a genetically engineered strain *Streptomyces avermitilis* MHJ1011. J Asian Nat Prod Res. (2017) 19:327–32. 10.1080/10286020.2016.1197911 27329378

[3] Fei CZ She RF Li GY Zhang LF Fan WS Xia SH. Safety and clinical efficacy of tenvermectin, a novel antiparasitic 16-membered macrocyclic lactone antibiotics. Eur J Pharm Sci. (2018) 117:154–60. 10.1016/j.ejps.2018.02.010 29427703

[4] Li G Cao X Liao J Wei Y. Pharmacokinetics of tenvermectin in swine, a novel antiparasitic drug candidate-comparison with ivermectin. Vet Med Sci. (2023) 9:1211–6. 10.1002/vms3.1085 36772910 PMC10188071

[5] Spatzenegger M Jaeger W. Clinical importance of hepatic cytochrome P450 in drug metabolism. Drug metab rev. (1995) 27:397–417. 10.3109/03602539508998329 8521748

[6] Thompson TN. Optimization of metabolic stability as a goal of modern drug design. Med Res Rev. (2001) 21:412–49. 10.1002/med.1017 11579441

[7] Trepaluier LA. Cytochrome P450 and its role in veterinary drug interactions. Vet Clin N Am-Small. (2006) 36:975–85. 10.1016/j.cvsm.2006.05.003 16984823

[8] Antonovic L Martinez M. Role of the cytochrome P450 enzyme system in veterinary pharmacokinetics: where are we now? Where are we going?. Future Med Chem. (2011) 3:855–79. 10.4155/fmc.11.37 21644832

[9] Fink-Gremmels J. Implications of hepatic cytochrome P450-related biotransformation processes in veterinary sciences. Eur J Pharmacol. (2008) 585:502–9. 10.1016/j.ejphar.2008.03.013 18417118

[10] Lin JH CYP. induction-mediated drug interactions: *in vitro* assessment and clinical implications. Pharm Res-dordr. (2006) 23:1089–116. 10.1007/s11095-006-0277-7 16718615

[11] Bogaards JJ Bertrand M Jackson P Oudshoorn MJ Weaver RJ van Bladeren PJ. Determining the best animal model for human cytochrome P450 activities: a comparison of mouse, rat, rabbit, dog, micropig, monkey and man. Xenobiotica. (2000) 30:1131–52. 10.1080/00498250010021684 11307970

[12] Hu SX Mazur CA Feenstra KL Lorenz JK Merritt DA. Assessment of inhibition of porcine hepatic cytochrome P450 enzymes by 48 commercial drugs. Vet J. (2016) 211:26–31. 10.1016/j.tvjl.2016.03.011 27053015

[13] Neodo A Schulz JD Huwyler J Keiser J. *In vitro* and *in vivo* drug-drug interaction study of the effects of ivermectin and oxantel pamoate on tribendimidine. Antimicrob Agents Chemother. (2019) 63:e00762-18. 10.1128/AAC.00762-18 30323047 PMC6325189

[14] Skalova L Szotakova B Machala M Neca J Lamka J Duchacek L. Effect of ivermectin on induction of cytochromes P450 in male rats. Chem Pap. (2000) 54:249−53. Available online at: https://chempap.org/?id=7&paper=2892

[15] Skalova L Szotakova B Machala M Neca J Soucek P Havlasova J. Effect of ivermectin on activities of cytochrome P450 isoenzymes in mouflon (*Ovis musimon*) and fallow deer (*Dama dama*). Chem-biol interact. (2001) 137:155–67. 10.1016/S0009-2797(01)00227-7 11551531

[16] Tan BS Opoku N Attah SK Awadzi K Kuesel AC Lazdins-Helds J. Pharmacokinetics of oral moxidectin in individuals with *Onchocerca volvulus* infection. PLoS Neglect Trop D. (2022) 16:e0010005. 10.1371/journal.pntd.0010005 35333880 PMC8986118

[17] Lewis DF Modi S Dickins M. Structure–activity relationship for human cytochrome P450 substrates and inhibitors. Drug Metab Rev. (2002) 34:69–82. 10.1081/DMR-120001391 11996013

[18] Le Bon AM Vernevaut MF Guenot L Kahane R Auger J Arnault I. Effects of garlic powders with varying alliin contents on hepatic drug metabolizing enzymes in rats. J Agric Food Chem. (2003) 51:7617–23. 10.1021/jf0346758 14664517

[19] Wang X Wang Y Wang Y Sun L. Gooneratne R. Preparation of T-2-glucoronide with rat hepatic microsomes and its use along with T-2 for activation of the JAK/STAT signaling pathway in RAW2647 cells. J Agric Food Chem. (2017) 65:4811–8. 10.1021/acs.jafc.7b01250 28556663

[20] Kumar S Samuel K Subramanian R Braun MP Stearns RA Chiu SHL. Extrapolation of diclofenac clearance from *in vitro* microsomal metabolism data: role of acyl glucuronidation and sequential oxidative metabolism of the acyl glucuronide. J Pharmacol Exp Ther. (2002) 303:969–78. 10.1124/jpet.102.038992 12438516

[21] Wang GF Hsieh Y Cui XM Cheng KC Korfmacher WA. Ultra-performance liquid chromatography/tandem mass spectrometric determination of testosterone and its metabolites in *in vitro* samples. Rapid Commun Mass Spectrum. (2006) 20:2215–21. 10.1002/rcm.2580 16791871

[22] Pegolo S Merlanti R Giantin M Dacasto M Montesissa C Capolongo F. High performance liquid chromatography determination of cytochrome P450 1A and 2C activities in bovine liver microsomes. Vet J. (2010) 183:81–8. 10.1016/j.tvjl.2008.08.006 18815059

[23] Tian X Cheng ZY He J Jia LJ Qiao HL. Concentration-dependent inhibitory effects of baicalin on the metabolism of dextromethorphan, a dual probe of CYP2D and CYP3A, in rats. Chem-Biol Interact. (2013) 203:522–9. 10.1016/j.cbi.2013.02.005 23458730

[24] Albassam AA Ahad A Alsultan A Al-Jenoobi FI. Inhibition of cytochrome P450 enzymes by thymoquinone in human liver microsomes. Saudi Pharm J. (2018) 26:673–7. 10.1016/j.jsps.2018.02.024 29989011 PMC6035319

[25] Kadian N Raju KSR Rashid M Malik MY Taneja I Wahajuddin M. Comparative assessment of bioanalytical method validation guidelines for pharmaceutical industry. J Pharmaceut Biomed. (2016) 126:83–97. 10.1016/j.jpba.2016.03.052 27179186

[26] Pekthong D Desbans C Martin H Richert L. Bupropion hydroxylation as a selective marker of rat CYP2B1 catalytic activity. Drug Metab Dispos. (2012) 40:32–8. 10.1124/dmd.111.041368 21965622

[27] Guengerich FP. Comparisons of catalytic selectivity of cytochrome P450 subfamily enzymes from different species. Chem-biol Interact. (1997) 106:161–82. 10.1016/S0009-2797(97)00068-9 9413544

[28] Jiang B Meng LY Zhang F Jin XL Zhang GL. Enzyme-inducing effects of berberine on cytochrome P450 1A2 *in vitro* and *in vivo*. Life Sci. (2017) 189:1–7. 10.1016/j.lfs.2017.09.011 28893642

[29] Xiao Y Xue X Wu YF Xin GZ Qian Y Xie TP. β-Naphthoflavone protects mice from aristolochic acid-I-induced acute kidney injury in a CYP1A dependent mechanism. Acta Pharmacol Sin. (2009) 30:1559–65. 10.1038/aps.2009.156 19890363 PMC4003015

[30] Minamiyama Y Takemura S Toyokuni S Imaoka S Funae Y Hirohashi K. CYP3A induction aggravates endotoxemic liver injury via reactive oxygen species in male rats. Free Radical Bio Med. (2004) 37:703–12. 10.1016/j.freeradbiomed.2004.05.022 15288127

[31] Li L Li ZQ Deng CH Ning MR Li HQ Bi SS. A mechanism-based pharmacokinetic/pharmacodynamic model for CYP3A1/2 induction by dexamethasone in rats. Acta Pharmacol Sin. (2012) 33:127–36. 10.1038/aps.2011.161 22212433 PMC4010279

[32] Hu N Huang YJ Gao XJ Li S Yan ZX Wei B. Effects of dextran sulfate sodium induced experimental colitis on cytochrome P450 activities in rat liver, kidney and intestine. Chem-Biol Interact. (2017) 271:48–58. 10.1016/j.cbi.2017.04.018 28438436

[33] Burke MD Thompson S Weaver RJ Wolf CR Mayer RT. Cytochrome P450 specificities of alkoxyresorufin O-dealkylation in human and rat liver. Biochem pharmacol. (1994) 48:923–36. 10.1016/0006-2952(94)90363-8 8093105

[34] Kobayashi K Urashima K Shimada N Chiba K. Substrate specificity for rat cytochrome P450 (CYP) isoforms: screening with cDNA-expressed systems of the rat. Biochem Pharmacol. (2002) 63:889–96. 10.1016/S0006-2952(01)00843-7 11911841

[35] Huang J Chen AL Zhang H Yu Z Li MH Li N. Gene replacement for the generation of designed novel avermectin derivatives with enhanced acaricidal and nematicidal activities. Appl Environ Microb. (2015) 81:5326–34. 10.1128/AEM.01025-15 26025902 PMC4510159

[36] Monbaliu J Gonzalez M Bernard A Jiao J Sensenhauser C Snoeys J. *In vitro* and *in vivo* drug-drug interaction studies to assess the effect of abiraterone acetate, abiraterone, and metabolites of abiraterone on CYP2C8 activity. Drug Metab Dispos. (2016) 44:1682–91. 10.1124/dmd.116.070672 27504016

[37] Canga AG Prieto AMS Liébana MJD Martínez NF Vega MS Vieitez JJG. The pharmacokinetics and metabolism of ivermectin in domestic animal species. Vet J. (2009) 179:25–37. 10.1016/j.tvjl.2007.07.011 17851096

[38] Wang X Cheung CM Lee WYW Or PMY Yeung JHK. Major tanshinones of Danshen Salvia miltiorrhiza exhibit different modes of inhibition on human CYP1A2, CYP2C9, CYP2E1 and CYP3A4 activities *in vitro*. Phytomedicine. (2010) 17:868–75. 10.1016/j.phymed.2010.05.003 20638257

[39] Zeng Z Andrew NW Woda JM Halley BA Crouch LS Wang RW. Role of cytochrome P450 isoforms in the metabolism of abamectin and ivermectin in rats. J Agric Food Chem. (1996) 44:3374–8. 10.1021/jf960222+

[40] Velík J Baliharová V Fink-Gremmels J Bull S Lamka J Skálová L. Benzimidazole drugs and modulation of biotransformation enzymes. Res Vet Sci. (2004) 76:95–108. 10.1016/j.rvsc.2003.08.005 14672851

[41] Alvarez L Lifschitz A Entrocasso C Manazza J Mottier L Borda B. Evaluation of the interaction between ivermectin and albendazole following their combined use in lambs. J Vet Pharmacol Ther. (2008) 31:230–9. 10.1111/j.1365-2885.2008.00953.x 18471144

[42] Szotáková B Baliharová V Lamka J Nozinová E Wsól V Velík J. Comparison of *in vitro* activities of biotransformation enzymes in pig, cattle, goat and sheep. Res Vet Sci. (2004) 76:43–51. 10.1016/S0034-5288(03)00143-7 14659728

[43] Martinez MN Antonovic L Court M Dacasto M Fink-Gremmels J Kukanich B. Challenges in exploring the cytochrome P450 system as a source of variation in canine drug pharmacokinetics. Drug Metab Rev. (2013) 45:218–30. 10.3109/03602532.2013.765445 23432217

[44] Jiang S Li H Zhang L Mu W Zhang Y Chen T. Generic Diagramming Platform (GDP): a comprehensive database of high-quality biomedical graphics. Nucleic Acids Res. (2025) 53:D16706. 10.1093/nar/gkae973 39470721 PMC11701665

