# Supplementary material for: Dose-dependent modulation of hepatic cytochrome P450 enzymes by tenvermectin: implications for medication safety and combination therapy
Source: Front Vet Sci. 2025 Aug 25;12:1647697. doi: 10.3389/fvets.2025.1647697 (PMC12415777; doi:10.3389/fvets.2025.1647697)
Supplement: Supplementary file 1 [file Supplementary_file_1.docx]

Supplementary Figures

**Dose-dependent modulation of hepatic cytochrome P450 enzymes by tenvermectin: implications for medication safety and combination therapy**

Jianping Liang^1,2^, Can Cui^2^, Wenge Ren^2^, Linglin Li^2^, Linyi Lv^2^, Xianhui Huang^1,2,^*, Xiangmei Li ^1,2,^*

^1^ College of Food Science, Guangdong Provincial Key Laboratory of Food Quality and Safety, South China Agricultural University, Guangzhou 510642; China

^2^ College of Veterinary Medicine, Guangdong Key Laboratory for Veterinary Drug Development and Safety Evaluation, South China Agricultural University, Guangzhou 510642, China

* Corresponding author: lixiangmei12@163.com; xhhuang@scau.edu.cn

**Figure captions**

**FIGURE S1** Related chromatograms of RF: (A) Water sample, (B) Blank liver microsome sample, (C) Matrix-spiked sample with 10 nmol/L RF, (D) Incubation sample with 5 μmol/L BR, (E) Incubation sample with 0.6 μmol/L ER.

**FIGURE S2** Related chromatograms of 4′-OH-DF: (A) Water sample, (B) Blank liver microsome sample, (C) Matrix-spiked sample containing 250 nmol/L 4′-OH-DF, (D) Matrix-spiked sample with 0.2 μmol/L IS, (E) Matrix-spiked sample with 250 nmol/L 4′-OH-DF and 0.2 μmol/L IS, (F) Incubation sample with 50 μmol/L DF.

**FIGURE S3** Related chromatograms of DOR: (A) Water sample, (B) Blank liver microsome sample, (C) Matrix-spiked sample with 1000 nmol/L DOR, (D) Incubation sample with 11 μmol/L DOM.

**FIGURE S4** Related chromatograms of 6β-OH-TS: (A) Water sample, (B) Blank liver microsome sample, (C) Matrix-spiked sample with 300 nmol/L 6β-OH-TS, (D) Matrix-spiked sample with 0.4 μmol/L IS, (E) Matrix-spiked sample with 600 nmol/L 6β-OH-TS and 0.4 μmol/L IS, (F) Incubation sample with 53 μmol/L TS.


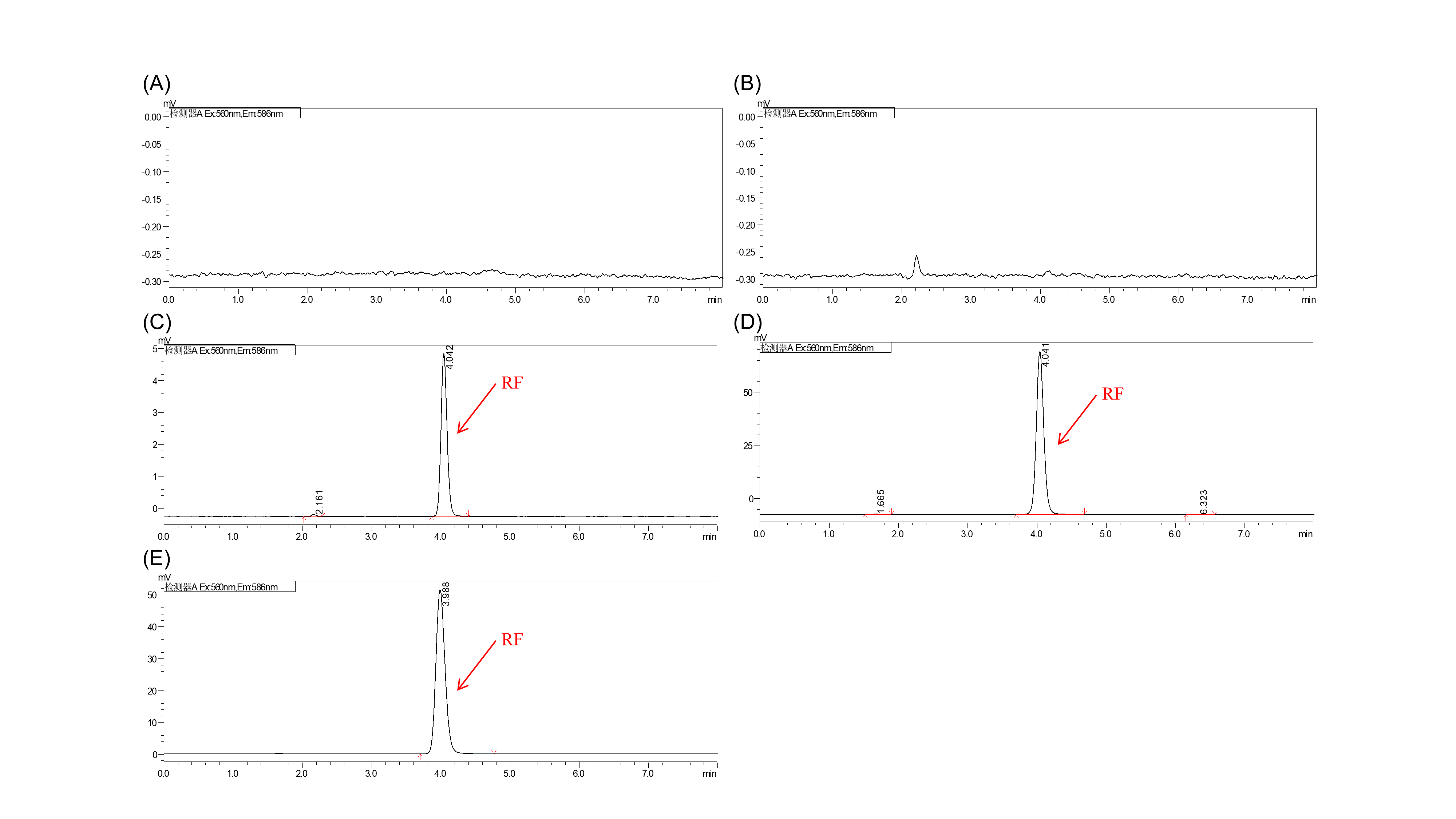


**FIGURE S1**


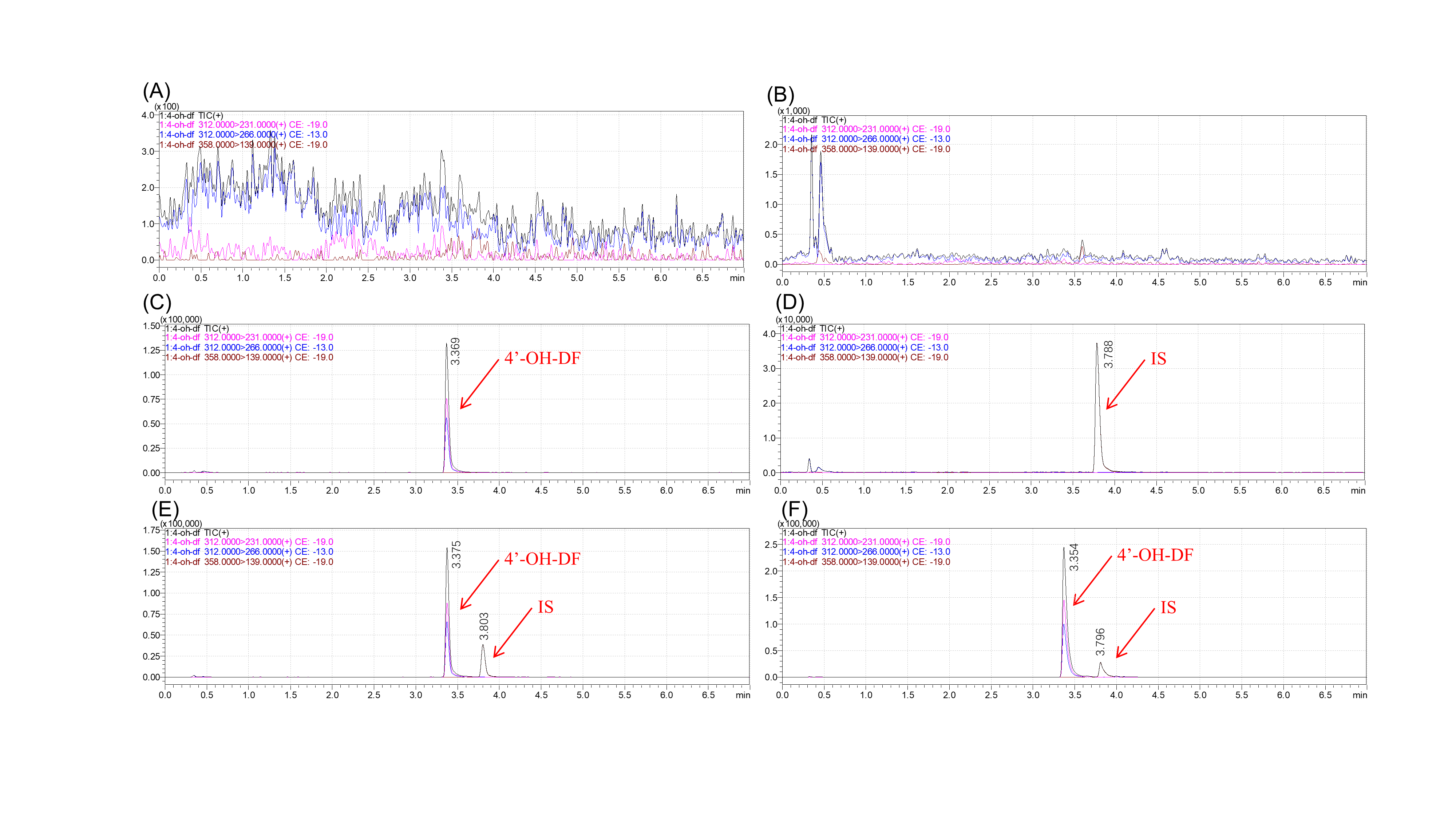


**FIGURE S2**


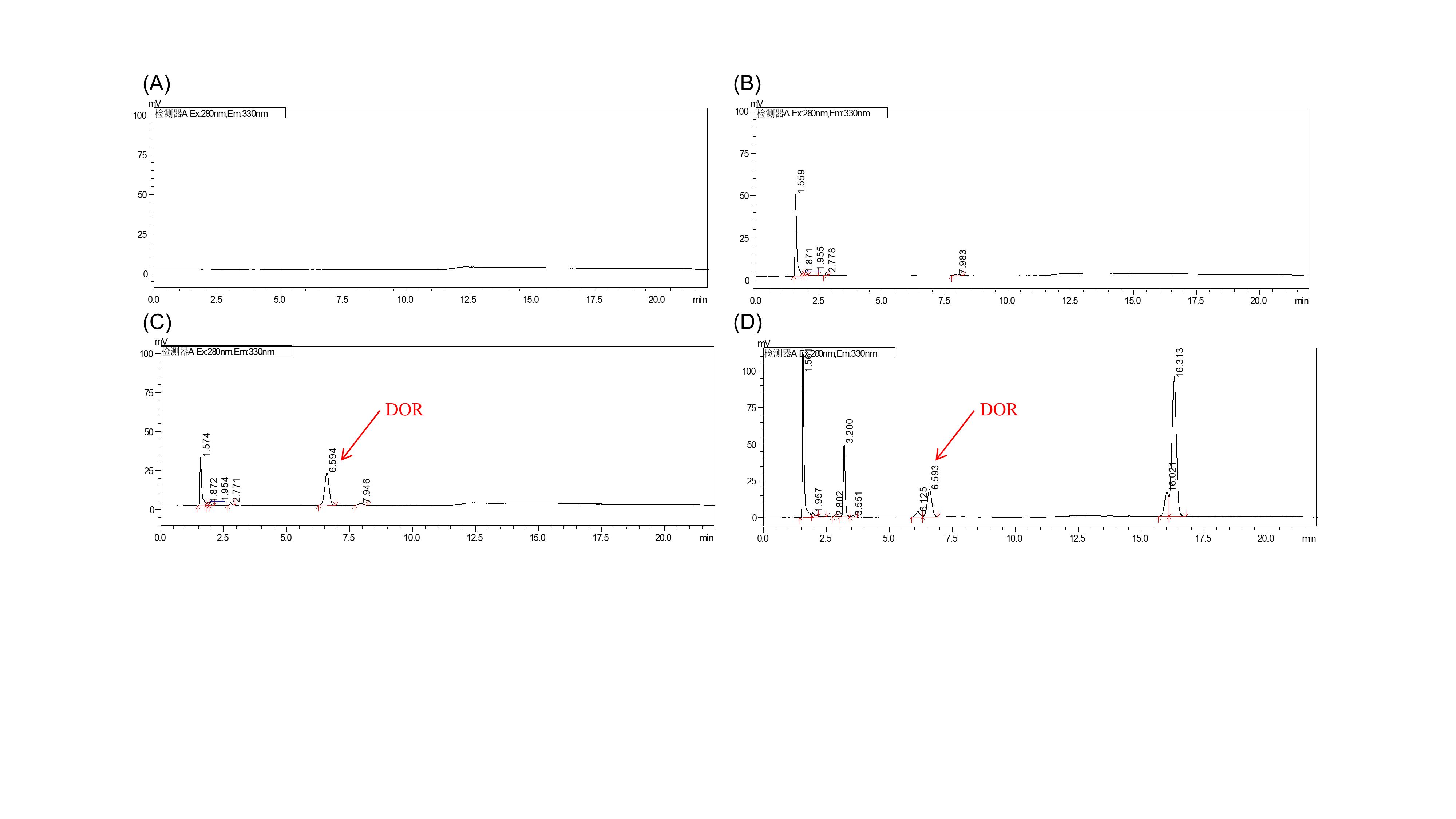


**FIGURE S3**


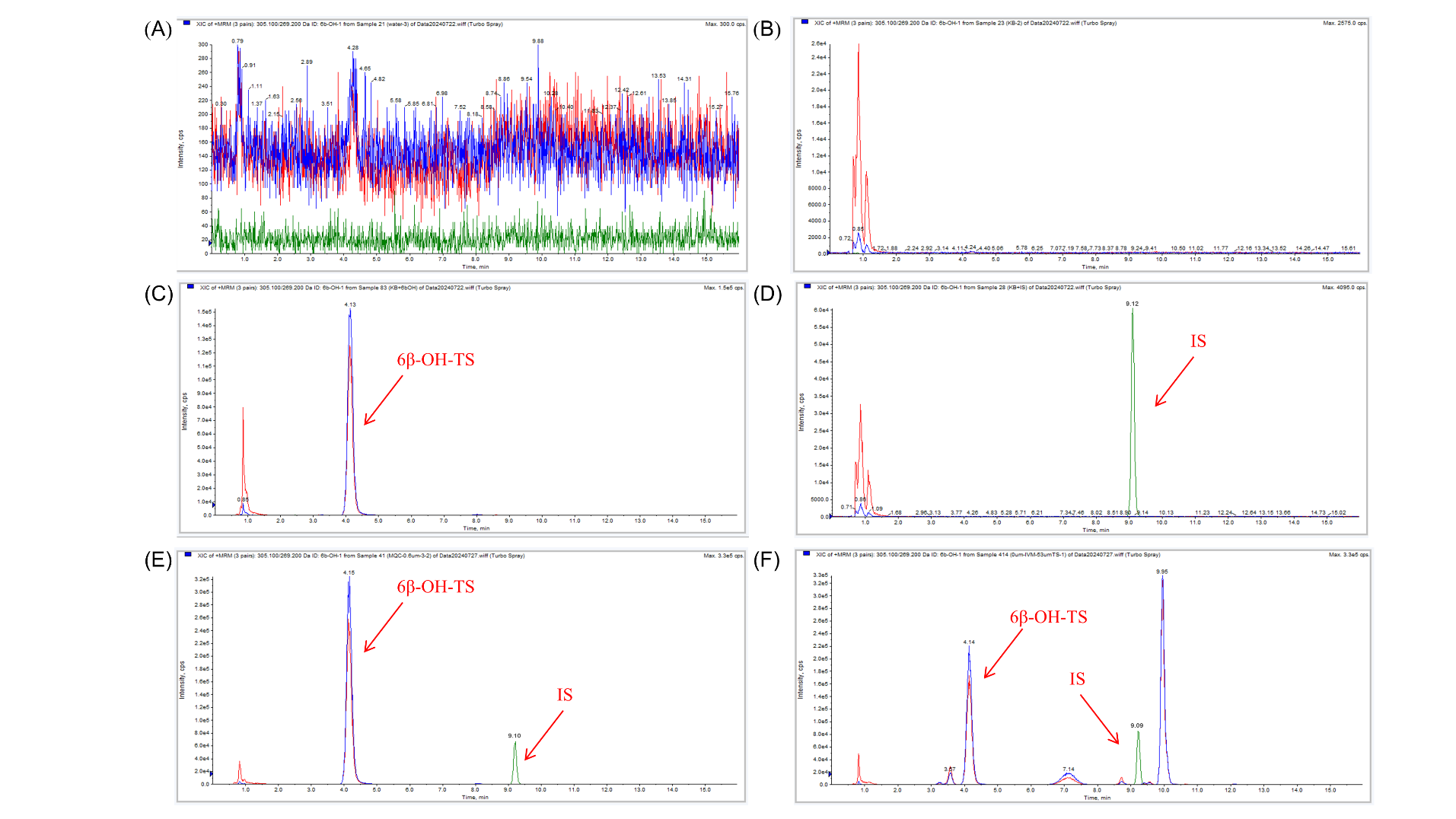


**FIGURE S4**
